# Growth status of first grader children in elementary schools in Watu Alo, Manggarai, East Nusa Tenggara

**DOI:** 10.1186/1687-9856-2015-S1-P35

**Published:** 2015-04-28

**Authors:** Dwi Lestari Pramesti, Srisadono Fauzi Adiwibowo, Komang Ferdiana, Ria Ramadhani, Hardian Gunardi, Aman Pulungan

**Affiliations:** 1 University of Indonesia, Jakarta, Indonesia; 2 Cipto Mangunkusumo Hospital, Jakarta, Indonesia

**Keywords:** children growth status, nusa tenggara

## Background

East Nusa Tenggara (NTT) had the highest number of children under five with mild to severe malnutrition in Indonesia, happened mostly in Manggarai Regency, with the highest number of cases found in Watu Alo Primary Health Care Supervision Area, with 5 out of all 19 cases in Manggarai, NTT (RISKESDAS, 2010). Nevertheless the method to classify the growth status was unclear.

## Objective

To determine growth status of first grader children in Watu Alo, Manggarai, NTT using the WHO and CDC growth charts and comparing the results.

## Method

A cross-sectional descriptive study. The data was taken from 19 to 22 August 2013 and 24 August 2013 in 5 elementary school under the supervision of Watu Alo Primary Health Care, Manggarai Regency, East Nusa Tenggara. Data interpretation with CDC and WHO growth chart.

## Results

CDC: the children are 74% short; 26% normal stature (median: 109 cm), 21.9% underweight; 69.4% normal weight; 6.1% overweight; and 2.6% obese (median: 18 kg). WHO: 72.4% of the children are normal stature; 20.9% stunted; 6.6% severely stunted, 84.7% normal weight; 6.6% risk of overweight; 6.6% wasted; 1% overweight; 0.5% obese; 0.5% severe wasted.

## Conclusions

To interpret the growth status, clinical judgement is also needed to be considered. Children growth status should be monitored continuously. Standardized national growth chart for Indonesian children should be made.

